# Exploratory testing of functional blood oxygenation level dependent-MRI to image the renoprotective effect of Remote Ischaemic PreConditioning during partial nephrectomy

**DOI:** 10.1038/s41598-024-83643-6

**Published:** 2024-12-30

**Authors:** Frederick Heuzeroth, Christian Wetterauer, Daniel Boll, Timm H. Westhoff, Maeve Dreher, Helge Seifert, Cyrill Rentsch, Jan Ebbing

**Affiliations:** 1 Department of Urology, University Hospital Basel, Basel, Switzerland; 2 Department of Radiology and Nuclear Medicine, University Hospital Basel, Basel, Switzerland; 3 Medical Department I, Marien Hospital Herne, University Hospital of the Ruhr-University Bochum, Herne, Germany

**Keywords:** Urology, Randomized controlled trials, Renal cancer, Magnetic resonance imaging

## Abstract

Urinary biomarker studies in cardiothoracic and kidney-sparing surgery have demonstrated renal protection by Remote Ischaemic PreConditioning (RIPC). RIPC intervention generates cycles of ischaemia and reperfusion of the limbs before the actual ischaemia of the target organ (e.g. kidney) is initiated. This explorative trial aims to investigate whether Blood Oxygenation Level Dependent-MRI (BOLD-MRI) can be a suitable technique to image and quantify the renoprotective effect of RIPC on ischaemia/reperfusion injury (IRI) after partial nephrectomy (PN). Overall, 15 patients were enrolled in this randomized controlled trial. Randomization was 1:1, with RIPC in the intervention arm. Urinary neutrophil gelatinase-associated lipocalin (NGAL), a sensitive biomarker for renal tubular damage was measured preoperatively and for the first 5 days after surgery. Functional BOLD-MRI was successfully performed preoperatively and 48 h after PN in 11 patients. BOLD-MRI uses ∆R2* to express acute tubular damage induced by IRI. The more the ∆R2* values have decreased postoperatively, the more damage the renal tubuli have taken. The cumulative urinary concentration of NGAL in the first 5 postoperative days was significantly lower in the RIPC group (p = 0.02) as compared to the control arm, indicating that the RIPC maneuver performed was effective. The highest difference was seen 6 h after surgery with NGAL being 65% lower in the RIPC arm. IRI of the operated kidney expressed by ∆R2* in BOLD-MRI was 2.1 times less pronounced in the RIPC group as compared to the noRIPC group (∆R2* in % preop/postop RIPC: 14.73/12.57 vs. noRIPC 16.33/11.82, p = 0.36). We were able to demonstrate the potential of BOLD-MRI in measuring IRI. For the first time, it was shown that the renoprotective effects of RIPC can be visualized and measured using BOLD-MRI. Larger studies are required to validate these initial findings.

## Introduction

About 25% of patients undergoing partial nephrectomy (PN) due to a renal mass already present with renal dysfunction with an estimated glomerular filtration rate (eGFR) of less than 60 ml/min/1.73m2 (chronic kidney disease (CKD) stage ≥ 3)^1,2^ and therefore are particularly at risk for further deterioration of renal function. Approximately 16%-40% of patients develop a CKD stage ≥ 3 due to PN^3,4^ and about 38%-40% suffer from acute kidney injury (AKI) immediately after surgery^5,6^. Initial kidney function and the amount of functional renal tissue removed are mainly responsible for the postoperative renal function and represent factors hard to influence. Intraoperative renal ischaemia on the other hand is an additional risk factor that needs to be controlled^6^. Intraoperative renal ischaemia induced by the surgeon by clamping major renal arteries, prevents severe bleeding but can be associated with impaired kidney function due to ischaemia/reperfusion injury (IRI) to the renal tubules^6,7^.

Cardiothoracic studies first demonstrated the reno- and cardioprotective potential of „Remote Ischaemic PreConditioning” (RIPC)^8–10^, significantly reducing myocardial injury and reducing infarct size^11^, aswell as lowering cardiac troponin levels and reducing major adverse cardiac events^12^​. There are also promising results using Ischaemic Conditioning for neuroprotective strategies and improved recovery in stroke patients^13^. RIPC intervention generates cycles of ischaemia and reperfusion of the limbs before the actual ischaemia of the target organ (e.g. kidney) is initiated. Ischaemia in RIPC is induced by inflating standard blood pressure cuffs on the arm and/or leg above the levels of the systolic blood pressure. The exact protective mechanism is not yet fully known. It is assumed that humoral and neuronal signalling cascades may be involved^14–16^. However, there is no consensus on the optimal duration and precise application of the preconditioning protocol itself, and the latency period after which RIPC exerts its beneficial effects.

There are only a few human studies that have investigated the renoprotective potential of RIPC in the setting of PN with IRI^17–21^. However, the use of biomarkers with low specificity, e.g. serum creatinine level, in the assessment of renal protection attributable to RIPC and/or the conduct of the experiment in the presence of two functioning kidneys compromised the interpretation and utility of their results. None of these studies ever used imaging to measure the immediate RIPC effect in the operated kidney.

Recently, we demonstrated that Blood Oxygenation Level Dependent-MRI (BOLD-MRI) can detect even mild tubular damage due to IRI in PN when a tubule-specific pharmacological manoeuvre is used^22^.

The aim of this explorative study is to evaluate the renoprotective potential of RIPC in patients undergoing PN using functional BOLD-MRI as a technical method for the quantitative visualisation of IRI-induced renal tubule damage. Our work is the first study to use this functional imaging trying to directly visualise and measure the potential renoprotective effect of RIPC on renal tubules. Furthermore, we measured the concentration of urinary neutrophil gelatinase-associated lipocalin (NGAL) as a well-studied, representative and sensitive marker of acute renal tubule damage^23^. In a previous study, we have already used PN with IRI as a model to investigate the dynamics of urinary biomarkers, including NGAL, indicative of acute renal tubule injury. Urinary NGAL has been shown to be a reliable and rapidly increasing marker for the detection of IRI-induced renal tubule damage, with low levels after IRI indicating little renal tubule damage and with urinary NGAL levels increasing within two hours of ischaemia^7^.

## Methods

### Study population

This study was approved by the local ethics committee “Ethikkommission Nordwest- und Zentralschweiz” (EKNZ Nr. 2017–01663) and was registered at ClinicalTrials.gov, Identifier: NCT03068689, first registration 03/03/2017. All participants provided written informed consent. All patients were scheduled for PN due to a renal mass at the Department of Urology of the University Hospital Basel and the research was performed in accordance with applicable guidelines and regulations. Exclusion criteria are shown in Table 1.

**Table 1:** Exclusion criteria and reasons for exclusion if an explanation appears necessary.

Exclusion criteria	Reason(s) for exclusion
Age < 18 years	
Pregnancy	
Significant peripheral arterial disease (PAD) affecting upper and/or lower limbs or history of PAD	It is unclear whether RIPC intervention worsens PAD
Significant renal disease CKD Stage 5 (GFR < 15 ml/min/1.73m2) or undergoing haemodialysis	It cannot be excluded that renal ischaemia in this constellation could be a relevant risk factor for accelerated and prolonged postoperative deterioration of renal function. Whenever possible, renal ischaemia should be avoided^6^ In the case of anuria, urine biomarkers cannot be tested
Concomitant therapy with glibenclamide or nicorandil	A potential interaction between these two drugs and RIPC has been described^10^
Concomitant therapy with non-steroid antirheumatics (NSAR), or other drugs known to impair renal function	
Use of propofol, except for induction of anaesthesia	The use of propofol in the context of total intravenous anaesthesia (TIVA) has been shown to interfere with the effects of RIPC^24^
Oral anticoagulation other than Acetylsalicylic acid (Aspirin) or International Normalized Ratio (INR) > 2	Risk of bleeding and risk of haematoma at the site of the blood pressure cuff

**Fig. 1:**
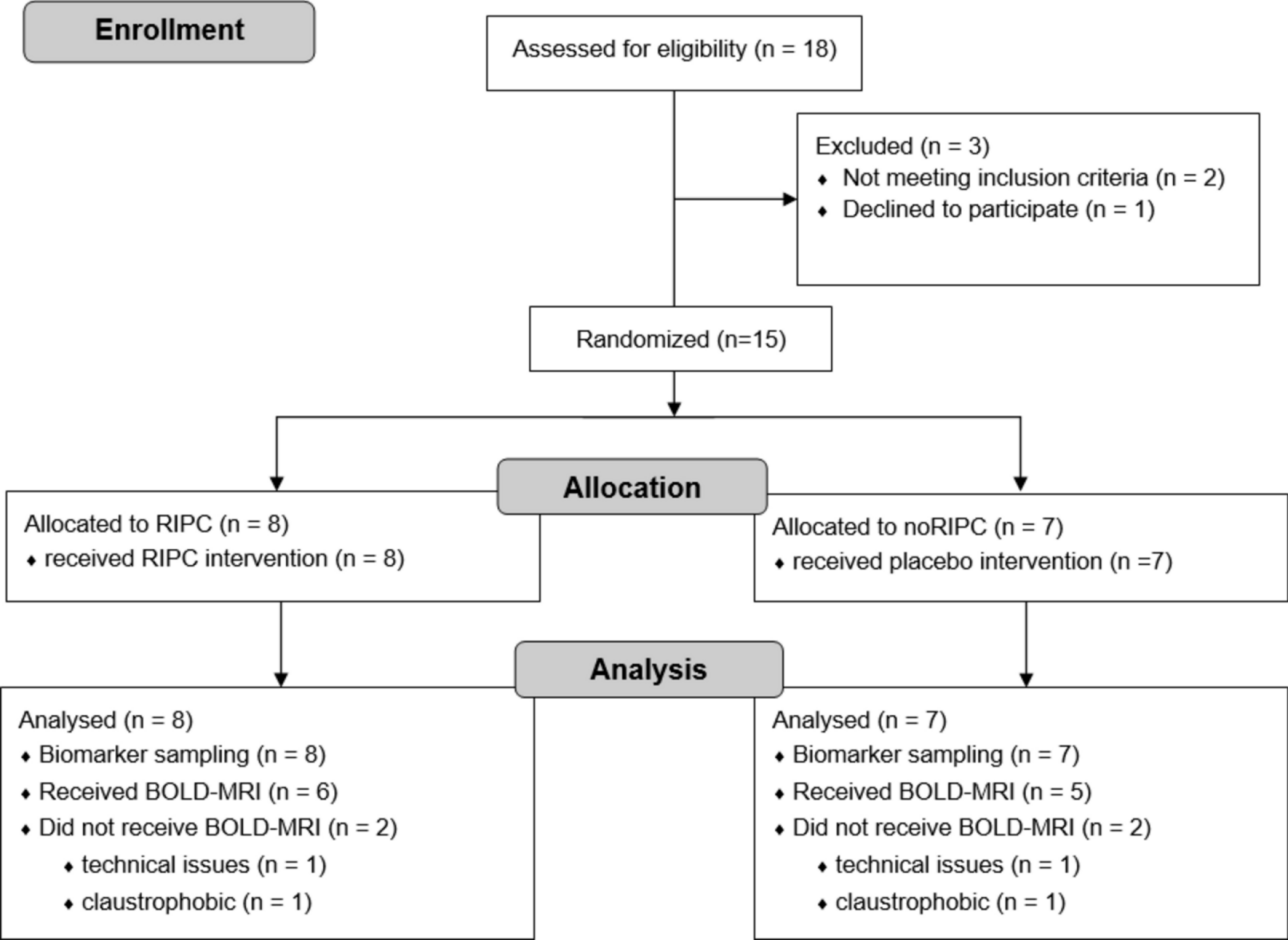
CONSORT flow diagram.

The study population comprised 15 patients randomised 1:1 into two groups. The intervention group received the RIPC procedure and the control group underwent a sham procedure (application of blood pressure cuffs without inflating them). The biomarkers of 15 patients were analysed (intervention group, n = 8 / control group, n = 7, Fig. 1), while BOLD-MRI data were only analysed for 11 patients due to the protocol deviations described in Fig. 1 which all occurred after randomization (intervention group, n = 6 / control group, n = 5, Fig. 1).

Epidemiological data, concomitant diseases, tumour characteristics, surgical data, functional renal parameters and urinary NGAL baseline concentrations are listed in Table 2. Kidney function is indicated by eGFR according to the Chronic Kidney Disease Epidemiology Collaboration (CKD-EPI) formula^25^ and by KDIGO (Kidney Disease: Improving Global Outcomes) CKD stages^26,27^. AKI is defined according to the Acute Kidney Injury Network (AKIN) criteria as an abrupt (within 48 h) increase in plasma creatinine levels ≥ 50% or ≥ 0.3 mg/dl^28^. Tumour complexity is described by R.E.N.A.L. Nephrometry Score^29^. The tumours were classified according to the TNM and WHO classifications for kidney tumours^30,31^.

**Table 2:** Patient characteristics.

	Intervention group (RIPC) (n = 8)	Control group (sham) (n = 7)	p-value
**Epidemiology**			
Male/female	6 (75%)/2 (25%)	5 (71%)/2 (29%)	0.99
Age (years)	53 (34–74)	61 (40–83)	0.16
Body mass index (kg/m2)	25.5 (20.7–35.5)	23.2 (18.2–28.1)	0.24
**Concomitant diseases**			
Diabetes mellitus	0 (0%)	1 (14%)	0.99
Hypertension	1 (12.5%)	4 (57.1%)	0.12
Pre-existing CKD (KDIGO G3a – G4)	0 (0%)	2 (28.5%)	0.20
Coronary heart disease	0 (0%)	1 (14%)	0.47
Status after nephrectomy	0 (0%)	0 (0%)	0.99
Status after NSS	0 (0%)	0 (0%)	0.99
**Medication on admission**			
ACE-I/ARB	1 (12.5%)	4 (57.1%)	0.12
Diuretics	1 (12.5%)	2 (29%)	0.57
**Side**			
Right/left kidney	5 (62.5%)/3 (37.5)	5 (71%)/2 (29%)	0.99
**Tumour characteristics**			
*Type of tumour*			0.47
RCC	8 (100%)	6 (86%)	
Oncocytoma	0 (0%)	0 (0%)	
Angiomyolipoma	0 (0%)	1 (14%)	
Other	0 (0%)	0 (0%)	
*RCC histology*			0.46
Clear cell	3 (37.5%)	4 (67%)	
Papillary	4 (50%)	2 (33%)	
Chromophobic	0 (0%)	0 (0%)	
Other	1 (12.5%)	0 (0%)	
*Staging of Renal Cell Cancer (RCC)*			0.43
pT1a	8 (100%)	5 (83%)	
pT1b	0 (0%)	0 (0%)	
pT2a	0 (0%)	1 (17%)	
pT2b	0 (0%)	0 (0%)	
Tumour diameter (mm)	22 (14–38)	25 (4–85)	0.98
R.E.N.A.L. Nephrometry Score	8 (6–8)	8 (4–9)	0.67
**Surgical data**			
Operation time (min)	215 (93–285)	165 (118–285)	0.35
Ischaemic time (min)	17.5 (12–22)	14 (9.45–20)	0.07
*Surgical approach*			0.2
Open surgery	0 (0%)	2 (29%)	
Endoscopic surgery	8 (100%)	5 (71%)	0.99
Robot-assisted laparoscopical	1 (12.5%)	1 (11%)	
Robot-assisted retroperitoneoscopical	7 (87.5%)	4 (89%)	
**Renal data**			
eGFR (CKD-EPI) on admission (ml/min/1.73m2)	97.8 (54.2–134.4)	66.5 (43.9–110.9)	0.34
Plasma creatinine on admission (mg/dl)	80.5 (50–129)	92 (46–129)	0.27
Urinary NGAL on admission (pg/ml)	12,382 (2631–48,389)	8985 (3007–74,917)	0.78

### Experimental intervention (RIPC)

The intervention treatment (RIPC) was performed with a standard blood pressure cuff on the upper arm and another standard blood pressure cuff on the thigh. The cuffs were simultaneously inflated to 200 mm HG and left inflated for 5 min., then deflated to 0 mm Hg and left uninflated for 5 min. This cycle was repeated so that the RIPC protocol lasted a total of 20 min. If systolic blood pressure was > 185 mm Hg, the cuffs were inflated to 15 mm Hg above this value. In the control group (sham), the cuffs were applied in the same way but not inflated. The RIPC protocol was started at the beginning of the first skin incision.

### Surgical technique

PN (nephron-sparing surgery, NSS) is the recommended gold standard in the treatment of patients with T1 renal cell cancer (RCC) or in patients with T2 RCC and a solitary kidney or CKD, if technically feasible^32^. In our study NSS was performed either by open surgery, robotic-assisted retroperitoneoscopic PN, or robotic-assisted laparoscopic PN. The decision whether to selectively clamp the main arteries, the segmental arteries or the tumour-supplying arteries was at the surgeon’s discretion. The number of vessels clamped and the ischaemia time were documented. Nephroprotective methods, such as induction of surface hypothermia of the kidney with ice slush or intravenous mannitol injections, were not applied.

### Functional BOLD-MRI

BOLD-MRT acquires multi-echo imaging sequences to determine the relaxivity of blood. The relaxivity of an MR contrast agent reflects how the relaxation rates of a solution change as a function of concentration.

**Fig. 2:**
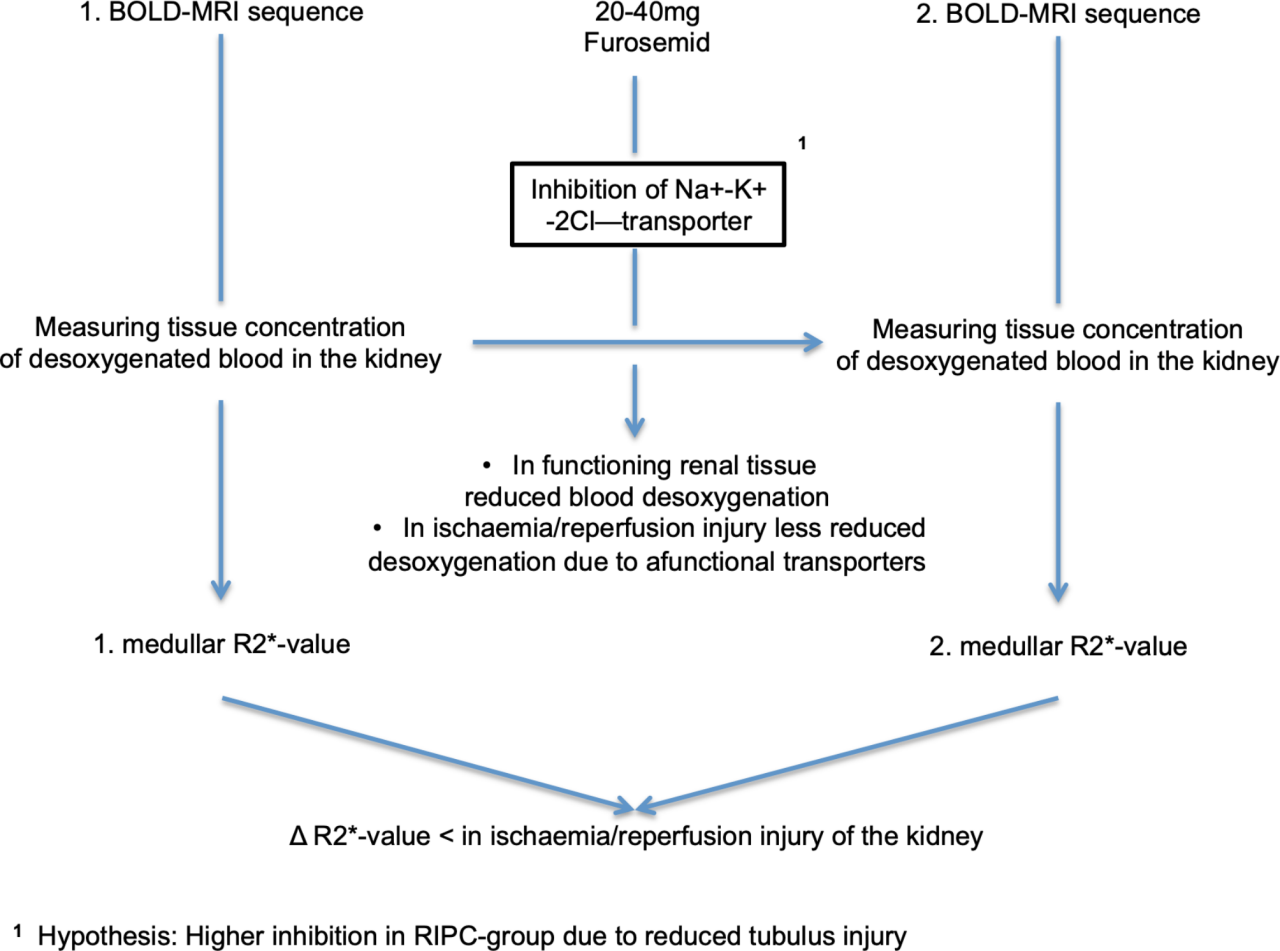
Functional BOLD-MRI procedure. BOLD-MRI procedure used with two sequences and a pharmacological manoeuvre using furosemide. The entire procedure is performed twice for each patient: once preoperatively and once on the second postoperative day.

BOLD-MRI is able to assess tissue oxygenation based on the transverse relaxation rate (R2*). R2* serves as a measure for deoxygenated hemoglobin. Our previously published data have shown that BOLD-MRI is an appropriate and non-invasive method to visualise IRI of the renal tubules in patients undergoing PN with renal artery clamping for renal tumours^22^. In order to test the function of the renal tubules, or to be able to quantify the extent of damage to the renal tubules by IRI, two sequences of BOLD-MRI must be performed, between which an intravenous application of 20–40 mg of furosemide is given. The first sequence shows and measures the tissue concentration of deoxygenated blood in the medulla of the renal parenchyma, called the medullary R2* value. Intrarenal medullary oxygen consumption depends mainly on the basal energy consumption of the tubules. Basal energy consumption is inhibited by administration of furosemide by blocking the Na–K-2Cl cotransporter. This leads to a decrease in the concentration of deoxygenated haemoglobin, which depends on the number of functional tubules. This means that the more functional tubules that can be inhibited, the greater the decrease in basal energy consumption. The second MRI sequence is performed after administration of furosemide to determine a second medullary R2* value, which allows the calculation of the ∆R2* value. In this way we can determine the changes in oxygen consumption before and after stimulation with furosemide. Based on the oxygen consumption, the functionality and damage of the kidney tissue can be determined. The more functionally intact tubules can be reduced in their oxygen consumption by furosemide, the greater the ∆R2* value; conversely, the more damaged channels cannot respond to furosemide, the less oxygen consumption is affected and the smaller the ∆R2* value (Fig. 2).

In other words, BOLD-MRI indicates IRI after NSS by the change in ∆R2* values. The more the tubules are damaged by IRI, the smaller the postoperative ∆R2* value compared to the preoperative ∆R2* value, so that the ∆ between preoperative and postoperative ∆R2* value increases. In the RIPC group, we expect more stable ∆R2* values from preoperative BOLD-MRI to postoperative BOLD-MRI. Therefore, the control group should have a larger ∆ from the pre- and postoperative ∆R2* values.

### Functional BOLD-MRI protocol

MRI examinations were performed preoperatively and 48 h after PN on a 1.5T magnetic resonance imaging system (Siemens Magnetom Avanto FIT) using 64-channel body phased array coils. The postoperative timing for the BOLD-MRI was chosen based on our experience with the dynamics of biomarkers for IRI after PN, indicating that the increase in urinary NGAL peaked on postoperative day 2. In addition, the data from the pilot study on BOLD-MRI were collected at median day 3 (range 2–5) after PN^22^.

Initially, three-plane spoiled gradient-echo localizers were performed. The clinical imaging protocol consisted of axial and coronal T2WI and axial T1WI in-phase and out-of-phase for a general anatomical overview, using a single-shot turbo spin-echo sequence for the T2WI images and spoiled gradient-echo sequences for the T1WI images. BOLD-MRI was performed during suspended respiration using a two-dimensional spoiled gradient-echo sequence with multiple echo time (TE) to obtain T2*-weighted images in the coronal plane. 8 echoes were obtained for each slice. For both of the two imaging time points in regard to PN, the BOLD-MRI was performed before and 7 min after administration of 40 mg furosemide. Furosemide was administered intravenously and flushed with 20 ml of saline. Our preliminary study has shown that the furosemide effect is most pronounced after 7 min. and is much more detectable in the medulla than in the cortex^22^. Table 3 summarizes the BOLD-MRI protocol.

**Table 3:** MRI protocol.

Parameter	BOLD
Orientation	Coronal
Repetition time (ms)	140
Echo times (ms)	3, 8, 13, 18, 23, 28, 33, 38
Flip angle (°)	40
Number of slices	3
Acquired matrix (pxl)	256 × 157
Reconstructed matrix (pxl)	512 × 314
Field of view (mm)	400 × 350
Percent phase encoding (%)	87.5
Slice sickness (mm)	5
Spacing between slices (mm)	13.5

### Analysis of MRIs and measurement of R2*

To determine the medullary R2* values, medullary regions of interest (ROI) were traced on the BOLD images. For each kidney, the slice showing optimal medullary differentiation, both before and after furosemide administration was selected. Multipoint ROIs were manually drawn on the first image of each BOLD series, which has proton density contrast, i.e. an anatomical representation of the tissues and negligible influence of BOLD effects due to the very short echo run time on the image T2*maps in the area of the medulla that were not obscured by artefacts. ROI measurements were subsequently cloned onto T2*maps acquired prior to and following furosemide administration. In three cases, the subsequent cloning of ROIs onto the T2* maps had to be corrected manually due to imaging artifacts caused by breathing movements. T2* images of the kidneys were calculated using Matlab (The Mathworks, Nattick, MA, USA) by fitting an exponential decay function on a voxel-by-voxel basis to the BOLD-MRI data. The signal intensity as a function of echo transit time gives the decay parameter T2* for each voxel. The T2* values and corresponding relaxation rates R2* were extracted for manually segmented ROIs for each series separately as the mean in that region and the error given by the standard deviation of these means.

### Biomarker sample collection

Urine samples were obtained for the assessment of urinary NGAL. NGAL receptors are found in the distal nephron where it mediates protein endocytosis^33^.It has been established as an early marker for AKI^23^. NGAL is released by acutely damaged tubular epithelial cells but does not reflect chronic tubular impairment in absence of ongoing tubular cell degradation^34^. We have great experience in measuring urinary NGAL in the context of kidney injury and have already intensively investigated the 5-day postoperative dynamics of urinary NGAL after PN with IRI compared to PN without IRI in a past study^7^.

The time points of collection were before surgery (baseline), 6 h postoperatively and once on each of 5 postoperative days. Blood creatinine was determined at the same time points. Collected urinary samples were stored at -20°C, until urinary biomarker concentrations were assessed. Concentrations of urinary NGAL were measured using the NGAL Rapid ELISA Kit (Bioporto, Gentofte, Denmark), according to manufacturer’s protocol as previously described^35^. This assay has been clinically validated^36^. An overview of all measurements over time is given in the following Table 4.

**Table 4:** Time points of biomarker collection and imaging.

	Baseline		6 h p.o	Day 1 p.o	Day 2 p.o	Day 3 p.o	Day 4 p.o	Day 5 p.o
Blood creatinine	x	Partial Nephrectomy	x	x	x	x	x	x
Urinary NGAL	x		x	x	x	x	x	x
BOLD-MRI	x				x			

### Statistical analysis

Continuous data are presented as median with interquartile range. In accordance with the results of a prioritised Shapiro–Wilk normality test and visual histogram analysis for normality, ordinal or continuous level data were analysed using the non-parametric Mann–Whitney test (e.g., levels of NGAL) and categorical parameter data were analysed using Fisher’s exact test (e.g., rate of acute kidney injury). A p-value of <0.05 was regarded as statistically significant. All statistical analyses were performed using GraphPad Prism 9.1.0 (GraphPad Software, Inc., La Jolla, CA, USA).

## Results

### Functional BOLD-MRI

Table 5 shows the pre- and postoperative results of R2* of the operated kidney before (baseline) and 7 min. after application of furosemide, the resulting absolute ∆R2* values and the ∆R2* values in percentage for the intervention group and the control group, respectively. ∆R2* values in percentage indicates that each R2* value before furosemide has been defined as 100 percent and has been set in relation to the corresponding R2* after furosemide thus generating a ∆R2* value in percentage points to maximize consideration of interindividual differences.

**Table 5:** R2* values raw data.

	Medullary R2* baseline	Medullary R2* 7 min after furosemid	Absolute ∆R2*	∆R2* in % from baseline
**Intervention group (RIPC)**				
Preop	0.016381697 (100%)	0.013777137 (84.1%)	0.00260456	15.9
	0.027593396 (100%)	0.024874362 (90.1%)	0.002719035	9.9
	0.036232447 (100%)	0.025969330 (71.7%)	0.010263117	28.3
	0.039619650 (100%)	0.036589830 (92.4%)	0.00302982	7.6
	0.017890797 (100%)	0.015466112 (86.4%)	0.002424685	13.6
	0.032964184 (100%)	0.026133373 (79.3%)	0.006830811	20.7
Postop	0.012698448 (100%)	0.012688546 (99.9%)	9.90274E-06	0.01
	0.023158787 (100%)	0.015499690 (66.9%)	0.007659097	33.1
	0.017517739 (100%)	0.016313480 (93.1%)	0.001204259	6.9
	0.017005261 (100%)	0.013898563 (81.7%)	0.003106697	18.3
	0.015251591 (100%)	0.012441538 (81.6%)	0.002810053	18.4
	0.013576367 (100%)	0.012989647 (95.7%)	0.000586721	4.3
**Control group (noRIPC)**				
Preop	0.013762267 (100%)	0.013442787 (97.7%)	0.00031948	2.3
	0.075585789 (100%)	0.062500000 (82.7%)	0.01308579	17.3
	0.015841800 (100%)	0.013255084 (83.7%)	0.002586716	16.3
	0.029074448 (100%)	0.022976304 (79.0%)	0.006098144	21.0
	0.015134436 (100%)	0.013439089 (88.8%)	0.001695347	11.2
Postop	0.012187429 (100%)	0.011791977 (96.8%)	0.000395452	3.2
	0.014455693 (100%)	0.012023199 (83.2%)	0.002432494	16.8
	0.015179545 (100%)	0.014778751 (97.4%)	0.000400793	2.6
	0.015872141 (100%)	0.013996624 (88.2%)	0.001875517	11.8
	0.05181347 (100%)	0.043725400 (84.4%)	0.00808807	15.6

**Fig. 3:**
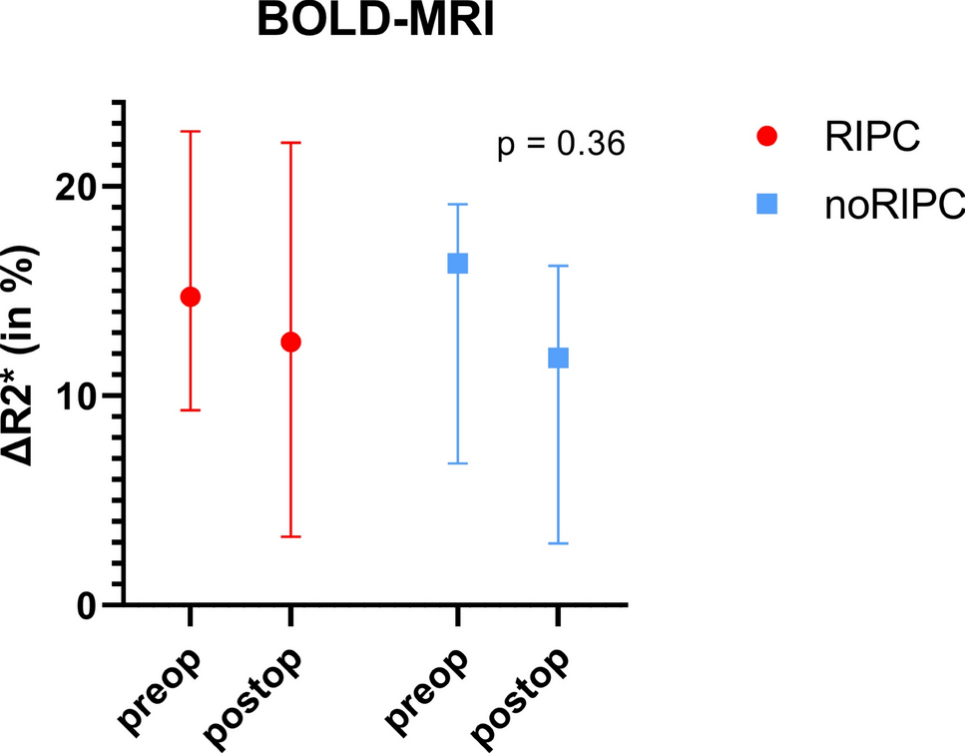
∆R2* (in %) before and after PN in the intervention group (RIPC) and control group (noRIPC). ∆R2* is presented in percentage points. The R2* before furosemide of each kidney has been defined as 100 percent and has been set in relation to the corresponding R2* after furosemide thus generating a ∆R2* value in percentage points to maximize consideration of interindividual differences. The lower the postoperative ∆R2* compared to the preoperative ∆R2*, the more renal tubules have been damaged by IRI.

While the RIPC group shows a slightly smaller ∆R2* (in %) after PN compared to before surgery (preop 14.73 (9.30–22.62) ∆R2* (in %) vs. postop 12.57 (3.26–22.09) ∆R2* (in %), p = 0.99), the control group shows a larger drop of the postoperative ∆R2* (in %), yet it is not statistically significant (preop 16.33 (6.76–19.14) ∆R2* (in %) vs postop 11.82 (2.94–16.22) ∆R2* (in %), p = 0.55), meaning that ∆R2* (in %) dropped 2.1 times less postoperatively in the RIPC group in contrast to the control group (∆R2* in % preop/postop RIPC: 14.73/12.57 vs. noRIPC 16.33/11.82, p = 0.36, Fig. 3). Remember, the lower the postoperative value of ∆R2* compared to the preoperative value of ∆R2*, the more damage the renal tubules have taken by IRI.

### Urinary NGAL biomarker

**Fig. 4:**
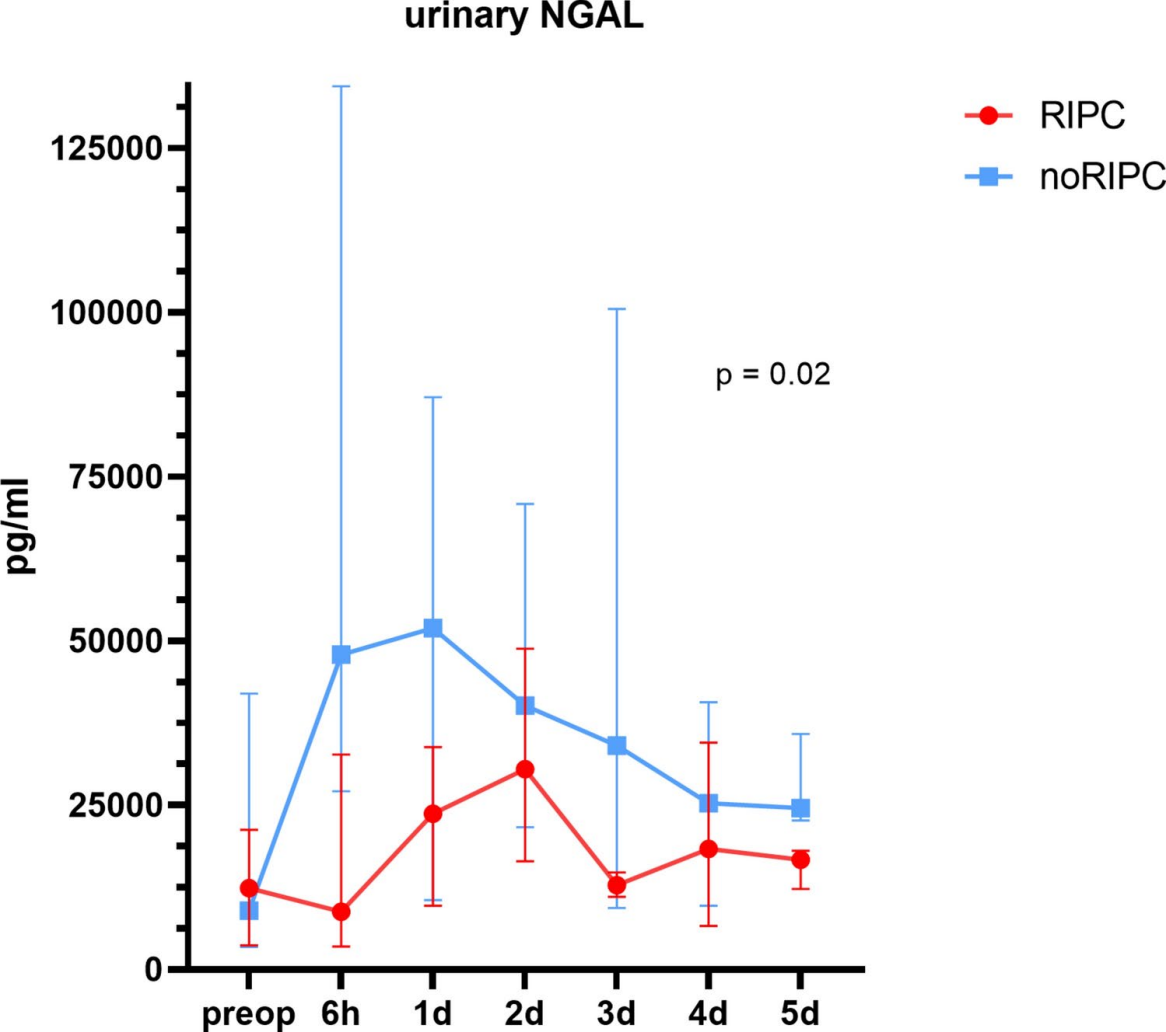
Pre- and postoperative concentrations of urinary NGAL for the intervention group (RIPC) and the control group (noRIPC). Preoperative (preop); days (d), hours (h).

Urinary NGAL showed a significant difference in cumulative levels over the first 5 postoperative days between the RIPC and noRIPC group, with a cumulative 39.6% lower NGAL concentration in the RIPC group (RIPC: 143,410 (95,239–179,989) pg/ml vs. noRIPC: 298,983 (154,657–392,290) pg/ml, overall total RIPC: 1,190,172 pg/ml vs. total noRIPC: 1,970,188 pg/ml, p = 0.02, Fig. 4). The greatest difference was recorded 6 h postoperatively, where NGAL was 65% lower in the RIPC group (RIPC: 8804 (4813–37,319) pg/ml vs. noRIPC: 47,936 (27,113–134,407) pg/ml, p = 0.04). On postoperative day 5, the difference between the two groups decreased to 12.1%, but was still statistically significant (RIPC: 16,720 (12,258–18,089) pg/ml vs. noRIPC: 24,577 (22,691–35,849) pg/ml, p = 0.03).

The rate of AKI was 25% (n = 2) in the intervention group and 57% (n = 4) in the control group, but without statistical significance (p = 0.31).

## Discussion

Remote ischemic conditioning (RIC) includes preconditioning, perconditioning, and postconditioning. Depending on the protective goal or type of intervention, the pre-, per-, or post-variant may be most suitable. The underlying mechanisms are essentially the same. For the following discussion, we will refer to them collectively as RIC. RIC has been extensively studied in the heart and brain, demonstrating at least preclinical protective effects against ischemia–reperfusion injury. In cardiology, RIC has been shown to reduce myocardial injury in myocardial infarcts^11^ and decrease cardiac troponin levels in patients undergoing coronary artery bypass graft surgery^12^. Similarly, in neurology, RIC has shown promise in reducing the extent of ischemic brain injury and improving functional outcomes after strokes. Clinical studies summarized in a review indicate that RIC can enhance cerebral blood flow, reduce infarct size, and promote neuroprotection through mechanisms like improved endothelial function and reduced inflammation, suggesting its potential as an adjunct therapy for stroke and other cerebrovascular events​^13^​. In general, RIC offers a non-invasive and practical approach to organ protection, with its benefits across different organ systems. The optimal method for performing RIC in routine clinical practice remains unclear, with at least seven different published protocols making it challenging to select the most suitable one^37^. Variations for instance in ischemia cycle durations, repetitions, pressure applications and blood cuff locations have not been systematically tested, and protocols are primarily based on empirical application. Some studies indicate that there are different protective time windows following a RIC procedure, with an acute window up to six hours after RIC and a chronic window offering protection 12 to 24 h after RIC^13^. However, there is a noticeable lack of standardized guidelines for conducting clinical RIC studies, which may contribute to the challenges in translating RIC into widespread clinical use. Addressing this significant gap requires identifying methods to distinguish between RIC responders and non-responders^38^. One noteworthy prospective study in cardiology demonstrated improved clinical outcomes by employing the lower limb for the RIC procedure, thereby involving a larger tissue mass^39^. This finding is corroborated by a mouse model study, which showed that the effect of RIC can be enhanced with a greater mass of ischemic/reperfused tissue^40^.

In recent years, only a limited number of human studies have investigated the presumed protective effect of RIPC on renal function in the context of renal surgery involving induced ischemia and reperfusion. These studies employed various designs, including different RIPC protocols, and utilized different target parameters to explore the common goal of "protection of the kidney." Different reviews and meta-analyses have shown mixed results, compiling data from studies with only secondary renal endpoints, varying definitions of AKI, and small sample sizes. While a potential effect of RIPC on renal function has been suggested, it has not been definitively proven. Further data is required to eliminate investigator-dependent bias. Once again, the lack of clear standardization hinders obtaining definitive answers^41–43^. Commonly, all these studies and reviews examined renal function based on plasma creatinine and eGFR or used specific biomarkers indicating acute renal damage due to tubular necrosis as target parameters. It is well understood that blood creatinine is not particularly well-suited as a sensitive marker for acute tubular damage caused by IRI, as it primarily reflects renal function and the loss of functional nephrons rather than renal injury^44^. Furthermore, IRI is not the sole factor affecting renal function after PN. For instance, the parenchymal volume preserved after PN has been shown to be an important factor in the preservation of eGFR^45^. Therefore, it is not surprising that different studies have reached varying conclusions regarding the role of RIPC in renal protection in the context of PN with renal ischemia. Additionally, unlike animal studies, human studies are complicated by the potential influence of the contralateral untreated kidney.

For a more precise assessment of renal injury, we measured urinary NGAL. NGAL is a biomarker for acute tubular damage and can be detected shortly after renal injury, making it a useful early indicator of AKI. Our present study confirms the results from our previous investigation of NGAL dynamics after PN with IRI^7^. However, while the previous study observed an NGAL peak on the second postoperative day, the control group in the current study exhibited a peak on the first postoperative day. Interestingly, the NGAL peak in the intervention group of our current study was not only significantly lower but also delayed, appearing on the second postoperative day. These results suggest that RIPC had a protective effect on the kidneys, as indicated by the lower NGAL values in the RIPC group. We provide a comprehensive picture of the dynamics of tubule damage under both non-RIPC and RIPC conditions due to an extended postoperative observation period. Other studies have also measured urinary NGAL levels to assess renal injury but with limited observation periods and mixed results. Hou et al. studied urinary NGAL levels two and six hours after PN in three groups: Group 1 received RIPC at the time of induction of anesthesia, Group 2 received RIPC 24 h before PN, and Group 3 served as the control group and did not receive RIPC. This study demonstrated a significantly lower increase in NGAL levels at both two and six hours in the RIPC groups compared to the control group^19^. Kil et al. presented the only study group that did not show an increase in urinary NGAL levels in either the RIPC or control group, despite ischemia times of 29 (17–51) minutes and 30 (14–38) minutes, respectively. Additionally, the baseline NGAL concentration was significantly lower in the RIPC group compared to the control group. Unfortunately, the authors were unable to provide a plausible explanation for these questionable results^18^.

However, none of the studies before used imaging techniques, particularly BOLD-MRI, to detect tubule damage. Our study addresses the question of whether and how well functional BOLD-MRI is able to reveal IRI-induced tubule damage, and whether BOLD-MRI can distinguish between different expressions of tubule damage under the same basic conditions when an influencing factor, such as RIPC, is introduced. Several techniques, including BOLD and diffusion-weighted imaging, have been used before to assess renal oxygenation or perfusion. The novelty of our current approach lies in combining BOLD-MRI with a tubule-specific pharmacological manoeuvre (blocking the Na + -K + -2Cl- cotransporter with furosemide) to gain specific information on tubular function. The pilot study on functional BOLD-MRI revealed that BOLD-MRI can detect even mild, subclinical IRI in the renal medulla in the context of PN^22^. The extent of tubular oxygen consumption depends on tubular integrity. Due to the activity of ATP-dependent transporters, tubular cells have exceptionally high oxygen consumption, resulting in a pO2 of only 10–15 mmHg even under physiological conditions. Consequently, the proportion of deoxygenated haemoglobin is high in the renal medulla. In acute tubular necrosis, however, tubular cells are unable to participate in reabsorption processes. Therefore, medullary concentrations of deoxygenated haemoglobin decrease, resulting in lower R2* values. Intrarenal R2* signals are influenced by the oxygen consumption of various cell types. To specifically examine oxygen extraction of tubular function, furosemide is administered as an inhibitor of energy-dependent electrolyte transport. If tubules are intact, furosemide leads to a decrease in deoxygenated haemoglobin and R2*. Thus, a lack of decrease in R2* after inhibition of the Na + -K + -2Cl- cotransporter is indicative of tubular damage, as indicated by a small ∆R2*^22^.

In contrast to the pilot study, this study also includes preoperative BOLD-MRI to directly compare ∆R2* values of the operated kidney with those before PN. Performing BOLD-MRI only postoperatively and comparing the ∆R2* values in each patient with the ∆R2* of the contralateral healthy kidney would likely have been too inaccurate, especially since the baseline kidney function in the control group, although not statistically significant, was nevertheless worse than in the intervention group. In the pilot study, the median ∆R2* after PN in the operated kidney was 8%, whereas in the contralateral kidney it was higher (20.7%), as expected. The kidneys in the healthy control group had a median ∆R2* of 30.7%. In the current study, both groups show similar preoperative ∆R2* values, with a slightly greater ∆R2* in the control group. Postoperatively, both groups show a decrease in ∆R2*, which can be plausibly explained by tubular damage. The finding that the decrease in ∆R2* in the RIPC group is 2.1 times less pronounced than in the control group suggests that BOLD-MRI has the potential to quantify the protective effect of RIPC on IRI-induced damage to the tubules, regardless of whether it is caused by necrosis or dysfunction. However, the difference is currently not statistically significant. Nonetheless, it has been shown that the application of RIPC significantly reduces NGAL levels. Knowing that the pharmacological BOLD-MRI maneuver targets the distal tubule by inhibiting the Na + -K + -2Cl- cotransporter, just as NGAL indicates damage to the distal tubule, it is reasonable to assume that the BOLD-MRI results reflect the statistically significant NGAL biomarker outcomes.

We are aware of several limitations to our study with the most significant being the small sample size of this explorative trial. The limited number of cases impacts the robustness of our findings. The absence of statistically significant difference in ∆R2* between the two groups is likely a direct result of this constraint. Originally, the BOLD-MRI analysis was planned to include 20 patients; however, we encountered recruitment difficulties, and due to institutional restrictions caused by a severe RIPC adverse event (unintended artificial ischemia of a patient’s limb for several hours resulting in a compartment syndrome), recruitment was ceased after enrolling 15 patients. Consequently, our findings should be considered purely exploratory, and further data are crucial to establish the statistical significance of BOLD-MRI in visualizing the nephroprotective effect of RIPC. Additionally, the small sample size has led to an uneven distribution in baseline characteristics, with the control group having poorer renal function compared to the intervention group. However, this does not substantially affect the interpretation of our results, as ΔR2*, in contrast to eGFR, is calculated separately for each kidney based on each patient’s individual baseline, and only the relative change with respect to the operated kidney and between the two groups (RIPC vs. non-RIPC) is compared. Furthermore, it is pertinent to acknowledge other biases, limitations and possible errors. The 11^th^ Hatter Cardiovasular Workshop^38^ defined four main categories of potential failure in RIPC application. 1. Signalling Failure: This phenomenon can occur in patients with co-morbidities such as diabetes or chronic renal impairment, due to reduced humoral release, neuropathy, or autonomic dysfunction. Considering the relatively young age of our study population compared to other RIC studies, signaling failure is not our primary concern. Additionally, the slightly higher prevalence of co-morbidities in the control group compared to the intervention group does not impact our findings, as the control group only underwent a sham RIPC procedure, and no signaling was expected in this group. 2. Target organ already protected: For example, via co-medication. Therefore, we excluded certain medications known to interfere with RIPC, such as platelet inhibitors and propofol^46^, but we cannot rule out the possibility that other medications may also interfere with RIPC^47^. 3. Study underpowered: This is likely given our sample size and the unknown effect size due to the exploratory nature of our study. 4. Target organ severely damaged: For instance, very poor renal function. This potential issue was mitigated by excluding patients with significant renal disease, defined as CKD Stage 5 (GFR < 15 ml/min/1.73m^2^) or those undergoing haemodialysis.

In addition, our study could not eliminate other potential confounders, such as variations in surgical technique among different surgeons, including parenchyma reconstruction, which might induce additional ischemia to the kidney. Furthermore, the ability to distinguish between IRI and postoperative kidney damage of a different origin remains a challenge. On the other hand, since no patient in the study received selective clamping, we can exclude this as a confounder. Additionally, the potential influence of resected peritumoral tissue on eGFR is excluded in the BOLD-MRI data, as the ROI used to calculate ∆R2* values focuses on the remaining kidney tissue. With respect to ischaemia times, the median duration of ischemia in the control group was 3.5 min shorter, potentially impacting the postoperative ∆R2* values. This implies that if the ischemia times were identical in both groups, the decrease in ∆R2* postoperatively could have been even more pronounced in the control group. In a recent investigation, we demonstrated that the risk of AKI following PN is related to the duration of ischemia and baseline renal function, and that patients with CKD stage ≥ 3a are vulnerable to kidney injury from the outset of ischemia^6^. In contrast, our previous study about NGAL dynamics in PN with IRI did not demonstrate a significant correlation between the duration of ischemia and the corresponding levels of NGAL. However, patients with CKD stage ≥ 3a were not included in this study^7^. It is reasonable to assume that the adverse impact of IRI on tubular function may be variable among individuals.

In summary, the present study investigates a combined approach of novel imaging techniques and biomarker measurements, with functional MRI imaging of IRI-induced renal tubule damage as the primary goal, supported by the quantification of urinary NGAL. The urinary NGAL measurements demonstrated the nephroprotective effect of our RIPC procedure. For the first time, we applied a functional BOLD-MRI imaging protocol that has the potential to visualize the renoprotective effect of RIPC. Our imaging approach involves measuring the difference in tubular oxygen consumption before and after the inhibition of an energy-consuming tubular transporter, which enables the quantification of intact tubular cells. This innovative methodology is particularly noteworthy, as it provides a novel means of visualizing, localizing, and quantifying tubular damage, thereby allowing for a more specific investigation of RIPC properties at the parenchymal level. The applied protocol could serve as a valuable tool for future studies aimed at analyzing damage or renoprotective effects in the distal tubule of the kidney. Further research with larger sample sizes is required to confirm the promising results of this exploratory trial and to correlate the non-significant ∆R2* findings with the significant biomarker results.

## Supplementary Information

Supplementary Information 1.

Supplementary Information 2.

## Data Availability

All data is available as Supporting Information S1.
